# Differences Between Indigenous Yeast Populations in Spontaneously Fermenting Musts From *V. vinifera* L. and *V. labrusca* L. Grapes Harvested in the Same Geographic Location

**DOI:** 10.3389/fmicb.2018.01320

**Published:** 2018-06-19

**Authors:** María L. Raymond Eder, Francisco Conti, Alberto L. Rosa

**Affiliations:** Laboratorio de Genética y Biología Molecular, IRNASUS-CONICET, Facultad de Ciencias Químicas, Universidad Católica de Córdoba, Córdoba, Argentina

**Keywords:** *Vitis*, *V. vinifera* L., *V. labrusca* L., grapes, indigenous yeast, fermentation

## Abstract

Yeast communities associated with *Vitis vinifera* L. ecosystems have been widely characterized. Less is known, however, about yeast communities present in grapes and fermenting musts from *Vitis* non-*vinifera* ecosystems. Moreover, there are no comparative studies concerning yeast communities in grapes from *V. vinifera* L. and non-*vinifera Vitis* species in vineyards from a shared *terroir*. In this work, we have used a culture-dependent strategy, phenotypic analyses, and molecular genotyping, to study the most representative yeast species present in spontaneously fermenting musts of grapes harvested from neighboring *V. vinifera* L. (cv. Malbec) and *V. labrusca* L. (cv. Isabella) vineyards. Phenotypic analyses of H_2_S production, ethanol tolerance and carbon utilization, on randomly selected strains of each *Hanseniaspora uvarum, Starmerella bacillaris* and *Saccharomyces cerevisiae* strains, as well as microsatellite genotyping of *S. cerevisiae* isolates from each the Malbec and Isabella grape musts, suggest that *V. vinifera* L. and *V. labrusca* L. ecosystems could harbor different yeast strain populations. Thus, microbial communities in exotic *Vitis* species may offer opportunities to look for unique yeast strains that could not be present in conventional *V. vinifera* L. ecosystems.

## Introduction

During alcoholic fermentation, a dynamic metabolic interaction between grape musts and their associated microbial communities shapes the final sensory and organoleptic character of wines (Fleet, 2008). Because of its scientific and industrial relevance, the study of the indigenous microbial communities in grapes and spontaneously fermenting grape must constitutes a major research area in oenology (Fleet, 2003; Jolly et al., 2014; Padilla et al., 2016; Varela, 2016; Varela and Borneman, 2017; Morgan et al., 2017). Culture-dependent and/or metagenomics approaches and DNA-based strategies have been used to characterize the complex and dynamic population of microorganisms in oenological ecosystems (Barata et al., 2012; Masneuf-Pomarede et al., 2016; Morgan et al., 2017). In these studies, a direct relationship has been recognized between grape microbiomas and *terroirs*, with the resulting specific microbial populations being a determining factor in the regional identity of vineyards, grapes, musts, and wines (Bokulich et al., 2014; Knight et al., 2015; Capece et al., 2016). A common pattern of development of yeast species, however, has been recognized in spontaneously fermenting musts from *Vitis vinifera* L. grapes, with non-*Saccharomyces* being the most common species at initial stages and *Saccharomyces* *cerevisiae* the dominant species at the middle and final stages of fermentation (Jolly et al., 2014). The rich diversity of non-*Saccharomyces* species, during the initial stages of fermentation, producing a variety of secondary metabolites, strongly contributes to the organoleptic signatures of wines (Jolly et al., 2006; Medina et al., 2013; Padilla et al., 2016; Varela, 2016).

While extensive research has been conducted on the complexity and dynamics of the yeast microbiota in the *V. vinifera* L. ecosystem (Varela and Borneman, 2017), fewer studies have examined the yeast communities in non-*vinifera Vitis* ecosystems. These non-conventional *Vitis* ecosystems may harbor a rich diversity of yeast species and strains (Raymond Eder et al., 2017). Recently, the diversity of yeasts in *V. labrusca* L. grapes and hybrids has been studied in vineyards from Brazil (Bezerra-Bussoli et al., 2013; Filho et al., 2017), the Azores Archipelago (Portugal) (Drumonde-Neves et al., 2016) and Argentina (Raymond Eder et al., 2017). These studies highlighted the remarkable diversity of non-*Saccharomyces* yeast species in a non-conventional *Vitis* ecosystem, and suggested the existence of specific *Vitis*-yeast species associations (Raymond Eder et al., 2017).

In this work, we report the identification and characterization of the main indigenous yeast species present during spontaneous fermentation of Malbec (*V. vinifera* L.) and Isabella (*V. labrusca* L.) grapes harvested from neighboring vineyards in Colonia Caroya (Córdoba, Argentina). Genetic and phenotypic characterization of a small number of isolates, representative of three relevant yeast species found in Malbec and Isabella ecosystems from this geographic region (i.e., *Hanseniaspora uvarum, Starmerella bacillaris*, and *S. cerevisiae*), suggest that spontaneously fermenting grape musts from different *Vitis* species could harbor different *Vitis*-specific yeast strain populations.

## Materials and Methods

### Spontaneous Fermentation of Malbec and Isabella Grape Musts

Malbec (*V. vinifera* L.) and Isabella (*V. labrusca* L.) grapes were harvested at their optimal ripeness stages from vineyards in Colonia Caroya (vintage of March, 2017), located at 31°02′00^′′^S / 64°05′36^′′^O and 491 meters above sea level, in the province of Córdoba, Argentina. The region has an annual rainfall of 765 mm and a mean temperature of 15.8°C. Separate spontaneous fermentations of a pool of destemmed and partially crushed Malbec and Isabella grapes were performed in a local cooperative cellar. Grapes from ∼80% of the Colonia Caroya’s Malbec and Isabella vineyards (i.e., 18–20 Ha each) are processed at this cellar. About 20% of these closely located, small vineyards (i.e., ∼1.5 Ha each), have intermixed rows of Malbec and Isabella plants. Must samples (70 liters) were fermented at 25–28°C in stainless steel tanks located in a room of the winery not previously used for winemaking. Musts were punched down twice a day and aliquots were taken daily for ten (i.e., 0–240 h) or five (i.e., 0–120 h) days from Malbec and Isabella musts, respectively, and stored in 30% (v/v) glycerol at -70°C.

### Isolation of Yeast Strains From Malbec and Isabella Ecosystems

Appropriate dilutions of fermenting Malbec and Isabella grape must samples were plated in duplicate on YPD-Cm agar [yeast extract 1.0% (w/v), peptone 2.0% (w/v), glucose 2.0% (w/v), agar 2.0% (w/v), chloramphenicol 10 μg/ml] and incubated for 5 days at 25°C. Colony counts on YPD-Cm plates were used to estimate the total number of yeast during fermentation. To identify the most predominant yeast species present at the initial stages of fermentation (i.e., 0, 24, and 48 h), 20 yeast colonies were randomly isolated from each sampling time from YPD-Cm agar plates having 30–50 independent colonies. These high dilution plates give a high probability of isolating strains belonging to dominant yeast species (Osorio-Cadavid et al., 2008; Raymond Eder et al., 2017). Additional colonies were randomly isolated from Malbec and Isabella musts at advanced stages of fermentation (i.e., 120 and 96 h, for Malbec and Isabella, respectively) and isolates identified as *S. cerevisiae* (i.e., 43 from Malbec and 32 from Isabella) were chosen for further analyses. Must samples from Malbec and Isabella, from early stages of fermentation (i.e., 0, 24 and 48 h), were also plated in duplicate on WL-Cm agar [WL Nutrient agar medium (Oxoid) 7.5% (w/v), chloramphenicol 10 μg/ml] and incubated for 5 days at 25°C. Ten yeast colonies from each of the Malbec and Isabella must samples analyzed (i.e., 0, 24, and 48 h) showing distinctive phenotypes (i.e., morphology and/or color), were isolated from these plates. These colonies could correspond to rare yeast species present at each sampling point (Raymond Eder et al., 2017). A total of 255 yeast isolates were obtained from the Malbec and Isabella ecosystems. All isolated yeasts were streaked on YPD agar, grown for 48 h at 25°C in YPD, and stored at -70°C in YPD broth with 30% (v/v) glycerol added.

### Molecular Identification of Yeast Species

Isolated yeasts were identified by PCR-RFLP and/or DNA sequencing of their 5.8-ITS (*Internal Transcribed Spacer*) rDNA regions (Esteve-Zarzoso et al., 1999). Total genomic DNA was extracted according to Raymond Eder et al. (2017). PCR was carried out using ITS1 and ITS4 primers (White et al., 1990). For PCR-RFLP, 10 μl of each of the PCR products were digested for 3 h at 37°C with the restriction enzymes *Hinf* I (New England BioLabs, United States) and/or *Cfo* I (Promega, United States) and the resulting DNA fragments were characterized by agarose [3.0% (w/v)] gel electrophoresis and analyzed using data from www.yeast-id.org. In most of the cases, yeast species identification was confirmed by Sanger sequencing of their 5.8-ITS rDNA regions and analysis using the BLASTN software NCBI^1^. Species identification was considered valid when the identity of a 5.8-ITS sequence and a reference sequence was 99–100%. Sequences from representative *H. uvarum, S. bacillaris*, and *S. cerevisiae* isolates were deposited in the NCBI GeneBank database (**Table 2**).

### Phenotypic Analyses of *H. uvarum, S. bacillaris*, and *S. cerevisiae* Isolates

Four random isolates of each *H. uvarum, S. bacillaris*, and *S. cerevisiae*, from each of the spontaneously fermenting Malbec and Isabella grape musts, were analyzed for production of H_2_S, ethanol tolerance, and fermentation ability in media containing either glucose or fructose as carbon sources. Control yeast strains used in these studies have been reported (Raymond Eder et al., 2017). H_2_S production was tested on Biggy-agar (*Bismuth Sulfite Glucose Glycine Yeast*; Oxoid). In these studies, 3 × 10^4^ cells (3 μl) were spotted on Biggy agar, incubated at 25°C for 3 days, and graded using the following visual color scale: 1 (white), 2 (cream), 3 (light brown), 4 (brown), and 5 (dark brown) (Sipiczki et al., 2001). Ethanol tolerance analyses were performed according to Belloch et al. (2008) with some modifications. Cells (3 × 10^4^ cells; 3 μl) were spotted on low dextrose [i.e., glucose 0.5% (w/v)] YP agar supplemented with either 0, 2.5, 5.0, 7.5, 10.0, 12.5, or 15.0% (v/v) of ethanol and incubated at 22°C. Growth was considered positive when colony development was recognized with the naked eye.

A simple weight loss microassay, dependent on CO_2_ release (Quirós et al., 2010), was designed to characterize glucose and fructose fermentation profiles of the *H. uvarum, S. bacillaris*, and *S. cerevisiae* isolates. Similar small scale fermentation assays have been recently published (Liccioli et al., 2011; Peltier et al., 2018). In our studies, strains were grown during ∼15 h at 25°C without agitation in 15 ml Falcon tubes containing 5 ml of YP medium supplemented with either glucose 10.0% (w/v) (YPD-10) or fructose 10.0% (w/v) (YPF-10). Duplicated 1.5 ml Eppendorf tubes, containing 1.0 ml liquid of YPD-10 or YPF-10, were inoculated with cells (10^7^/ml) from the YPD-10 and YPF-10 cultures, respectively, and maintained at 25°C without agitation. Microtubes contained a 0.8 mm perforation on its cap, covered with a small piece of cotton, to allow CO_2_ efflux. Tubes were weighed immediately after inoculation and every 24 h for 4 days, using non-inoculated tubes as control of weight loss via evaporation. Fermentation rates were expressed as weight loss (i.e., CO_2_ release) in function of time (i.e., g.l^-1^.h^-1^).

### *S. cerevisiae* Microsatellite Genotyping

*Saccharomyces cerevisiae* isolates from Malbec and Isabella musts were genotyped using seven microsatellite loci (i.e., SCAAT1, SCAAT2, SCAAT3, C3, C6, YPL009c, and SCYOR267c) (Legras et al., 2005). PCR reactions contained 100 ng of genomic DNA, 1.5 mM MgCl_2_, *Taq* polymerase buffer 1X (Invitrogen, United States), 200 μM dNTPs, 10 pmol of each primer and 1.25 units of *Taq* polymerase (Invitrogen, United States). Amplification reactions were performed in a MJ Mini Bio-Rad thermocycler (Bio-Rad, United States) using an initial denaturation step at 95°C for 5 min, followed by 35 cycles of 95°C for 30 s, annealing at 57°C for 45 s, extension at 72°C for 1 min followed by a final extension at 72°C for 10 min. PCR products were separated in 8.0% polyacrylamide gels using TBE as the running buffer. Gels were stained with ethidium bromide, photographed under UV light and allele sizes were determined using the *100-bp-DNA-ladder* (Inbio Highway, Argentina) as a reference molecular size standard.

## Results

### Malbec (*V. vinifera* L.) and Isabella (*V. labrusca* L.) Spontaneously Fermenting Grape Musts

Standard oenological analyses of Malbec and Isabella grape musts were performed at the beginning and the end of fermentation (**Table 1**). As expected, from its low initial levels of total reducing sugars, Isabella grape must fermentation was completed in 5 days, while spontaneous fermentation of Malbec grape must took 10 days. Ethanol concentration in completely fermented Isabella grape must was 8.9% (v/v), which is ∼1% (v/v) lower than expected from its initial concentrations of reducing sugars (169.5 g/l) (**Table 1**).

**Table 1:** Physicochemical analyses of spontaneously fermenting Malbec and Isabella grape musts.

Parameter	Malbec (days)		Isabella (days)	
	0	10	0	5
Reducing sugars (g/l)	226.0	2.20	169.5	1.8
Ethanol (%)	0	13.3	0	8.9
Acidity (tartaric acid) (g/l)	5.40	5.25	6.90	6.97
PH	3.90	3.94	3.42	3.43

### Population Dynamics and Main Cultivable Yeasts in Spontaneously Fermenting Malbec and Isabella Grape Musts

**FIGURE 1:**
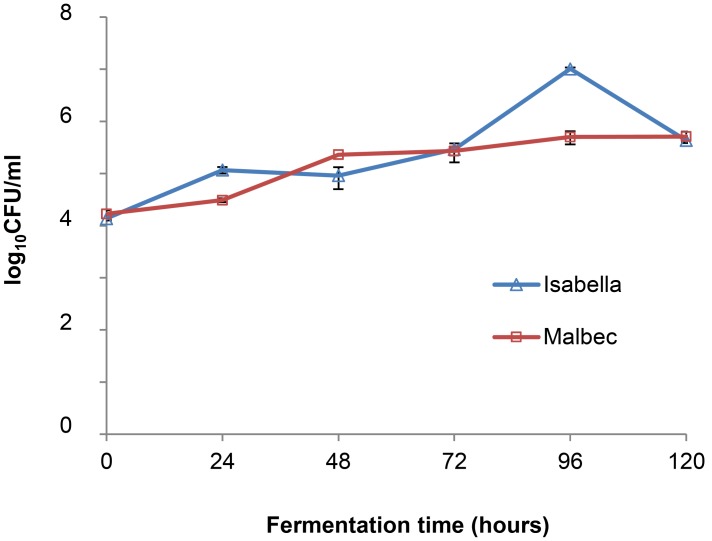
Population dynamics of total yeasts at initial times of spontaneous fermentation of Malbec and Isabella grape musts.

**FIGURE 2:**
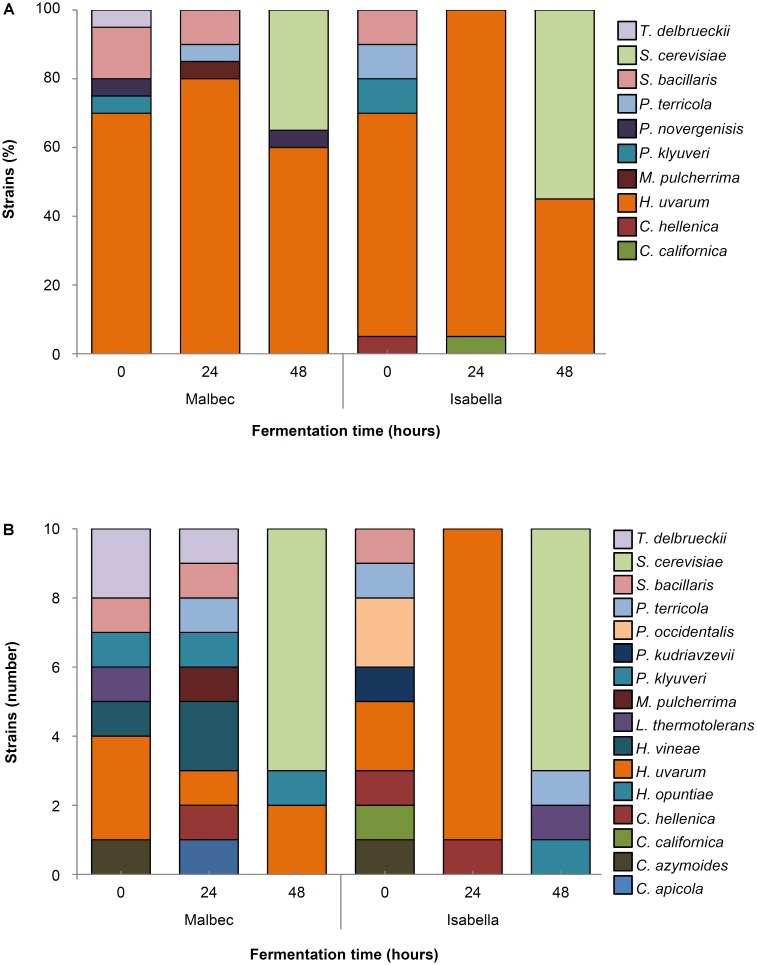
Main contributing yeast species during spontaneous fermentation of Malbec and Isabella grape musts. Percentages represent the relative contribution of the indicated yeast species among 120 randomly selected colonies (20 isolates/sampling time; 60 isolates from each Malbec and Isabella) obtained at the indicated times of fermentation **(A)**. Yeast species identified among 60 rare colonies (10 isolates/sampling time; 30 isolates from each Malbec and Isabella) isolated from WL-Cm Nutrient agar plates at the indicated times of fermentation **(B)**.

The population dynamics of cultivable yeast species in the Malbec and Isabella ecosystems were analyzed from time *t0* to *t120*, corresponding to the initial stages of fermentation of Malbec and the entire fermentation period of Isabella (**Figure 1**). The total yeast populations in both ecosystems started with similar counts, and increased similarly as fermentation progressed (**Figure 1**). The highest total yeast count in fermenting Isabella grape must was observed at *t96* while fermenting Malbec must reached its highest yeast count at *t120* (**Figure 1**). As expected, *S. cerevisiae* was the most predominant yeast species recognized among 75 isolates obtained at the middle/advanced stages of fermenting Malbec and Isabella musts (i.e., *t72*–*t120*) (not shown). Based on this observation, our analyses of the predominant non-*Saccharomyces* species in the Malbec and Isabella ecosystems were limited to the early stages of spontaneous fermentation (i.e., *t0, t24*, and *t48*), at which a total of 180 isolates were identified by PCR-RFLP and/or DNA sequencing of their 5.8-ITS (*Internal Transcribed Spacer*) rDNA regions (**Figure 2**).

A great diversity of non-*Saccharomyces* species was evidenced among the yeast isolated from both Malbec and Isabella ecosystems (**Figure 2**). *H. uvarum* was the most common species isolated at early stages of fermentation of Malbec (*t0, t24*, and *t48*) and Isabella (*t0* and *t24*) grape musts (**Figure 2A**). Other non-*Saccharomyces* yeast species identified in both Malbec and Isabella musts were *Candida azymoides, Candida hellenica, Lachancea thermotolerans, Pichia klyuveri, Pichia terricola*, and *Starmerella bacillaris*. Interestingly, *Torulaspora delbrueckii, Hanseniaspora vineae*, and *Metschnikowia pulcherrima*, were not among the yeast species identified in Isabella (**Figure 2**). *Candida californica*, previously recognized in Isabella fermenting must (Raymond Eder et al., 2017), was also isolated from Isabella in this work (i.e., at *t0* and *t24*). *P. occidentalis* and *P. kudriavzevii* were isolated only from fermenting Isabella must (**Figure 2B**) while *P. norvegensis* was isolated only from fermenting Malbec must. *S. cerevisiae*, not isolated at initial stages of spontaneous fermentation, was the predominant yeast species in Isabella must at *t48*, and started to become dominant at the same fermentation time in Malbec must.

### H_2_S Production by *H. uvarum, S. bacillaris*, and *S. cerevisiae* Isolates From the Malbec and Isabella Ecosystems

**FIGURE 3:**
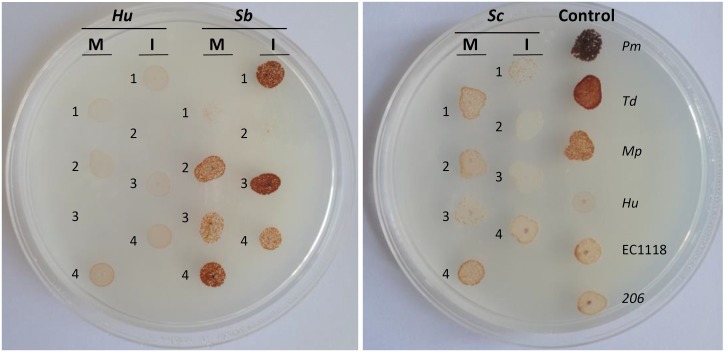
H_2_S production. Isolates 1 to 4 from each *H. uvarum* (*Hu*), *S. bacillaris* (*Sb*), and *S. cerevisiae* (*Sc*) (**Table 3**), from the Malbec (*M*) and Isabella (*I*) ecosystems, as well as control strains *P. membranifaciens* (*Pm*), *T. delbrueckii* (*Td*), *M. pulcherrima* (Mp), *H. uvarum* (*Hu*), and *S. cerevisiae* (*EC1118* and *206*), were grown in Biggy medium and H_2_S production was scored (see **Table 2**) as indicated in the Material and Methods section.

In order to explore possible phenotypic differences among *S. bacillaris, H. uvarum*, and *S. cerevisiae* isolates from the Malbec and Isabella ecosystems, we analyzed the production of H_2_S in four randomly selected isolates from each of these species. H_2_S production varied greatly among yeast species, as well as between isolates of the same species from the same ecosystem (**Figure 3** and **Table 2**). Interestingly, however, most of the *S. cerevisiae* isolates from Malbec (3 out of 4) showed higher production of H2S than their counterparts isolated from Isabella grape must (**Figure 3**). *S. bacillaris*, on the other hand, was the species with the most consistent production of relatively high levels of H_2_S, compared to *H. uvarum* and *S. cerevisiae* (**Figure 3** and **Table 2**).

**Table 2:** Phenotypic analyses of *H. uvarum, S. bacillaris*, and *S. cerevisiae* isolates from neighboring Malbec and Isabella ecosystems.

Species	Isolate	Strain^1^	H_2_S^2^	Ethanol (%)^3^	Genbank
*H. uvarum*	1	MT017-035	2	2.5	MG734841
	2	MT117-032	2	2.5	MG734842
	3	MT217-024	1	2.5	MG734843
	4	MT217-031	3	2.5	MG734844
	1	IT017-034	2	2.5	MG734838
	2	IT117-025	1	2.5	MG734837
	3	IT117-013	2	2.5	MG734839
	4	IT217-014	2	2.5	MG734840
*S. bacillaris*	1	MT017-001	2	2.5	MG734849
	2	MT017-005	3	2.5	MG734850
	3	MT117-001	3	2.5	MG734851
	4	MT217-002	4	2.5	MG734852
	1	IT017-025	4	5.0	MG734845
	2	IT017-033	2	2.5	MG734846
	3	IT017-051	4	2.5	MG734847
	4	IT217-001	3	2.5	MG734848
*S. cerevisiae*	1	MT217-023	3	10.0	MG734853
	2	MT317-003	3	10.0	MG734854
	3	MT417-002	2	10.0	MG734855
	4	MT517-001	3	10.0	MG734856
	1	IT217-022	2	12.5	MG734858
	2	IT217-029	1	10.0	MG734857
	3	IT317-004	1	12.5	MG734859
	4	IT517-004	2	12.5	MG734860
*Pm*		RG02	5	12.5	Ref^4^
*Td*		RG07	4	10.0	Ref^4^
*Mp*		RG01	3	5.0	Ref^4^
*Hu*		RG06	2	2.5	Ref^4^
*Sc*		EC1118	3	12.5	Ref^4^

*^1^M (Malbec) and I (Isabella) strains; ^2^H_2_S production was evaluated in Biggy medium and scored as indicated in the Material and Methods section; ^3^Tolerance to ethanol is indicated as the maximal ethanol concentration [i.e., % (v/v)] in solid media where strain growth was observed. ^4^Raymond Eder et al. (2017). Pm (P. membranifaciens), Td (T. delbrueckii), Mp (M. pulcherrima), Hu (H. uvarum), and Sc (S. cerevisiae).*

### Ethanol Tolerance of *H. uvarum, S. bacillaris*, and *S. cerevisiae* Isolates From the Malbec and Isabella Ecosystems

Tolerance to ethanol of the 24 randomly selected *H. uvarum, S. bacillaris*, and *S. cerevisiae* isolates was determined according to their ability to grow in solid media supplemented with different concentrations of ethanol (i.e., 2.5–15.0%). In these studies, *S. bacillaris* isolates from both Malbec and Isabella ecosystems were able to grow only in media containing relatively low levels of ethanol (i.e., 2.5 to 5.0%) (**Table 2**). Most of the *S. cerevisiae* isolates from Isabella (3 out of 4) showed higher ethanol tolerance (i.e., 12.5%) than the four characterized *S. cerevisiae* isolates from Malbec must (i.e., 10.0%). The relatively low tolerance to ethanol of the Malbec *S. cerevisiae* isolates was also observed in *S. cerevisiae* isolates from more advanced stages of fermentation of the Malbec must (not shown). Similar results were obtained when ethanol tolerance of the *H. uvarum, S. bacillaris*, and *S. cerevisiae* isolates was assayed in liquid media (not shown).

### Fermentation Profiles of *H. uvarum, S. bacillaris*, and *S. cerevisiae* Isolates From the Malbec and Isabella Ecosystems

**FIGURE 4:**
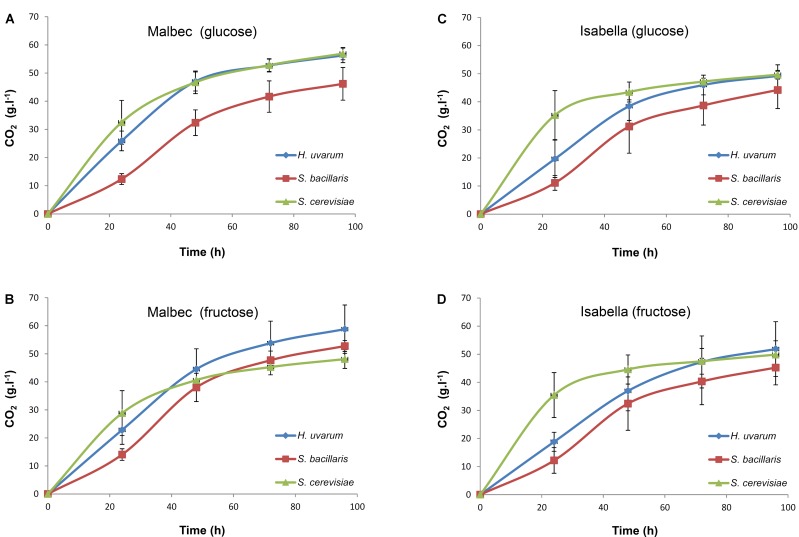
Fermentation profiles of *H. uvarum, S. bacillaris*, and *S. cerevisiae* isolated from the Malbec and Isabella ecosystems. Average weight loss (i.e., CO_2_ release) of 1.0 ml cultures of the Malbec **(A,B)** and Isabella **(C,D)** *H. uvarum, S. bacillaris*, and *S. cerevisiae* isolates, indicated in **Table 2**, grown during 96 h in media containing 10% (w/v) glucose **(A,C)** or 10% (w/v) fructose **(B,D)** as carbon sources. Each point represents the average value (i.e., expressed as g.l^-1^.h^-1^) of eight independent cultures (i.e., duplicate cultures of the four isolates tested for each species) ± SD.

**FIGURE 5:**
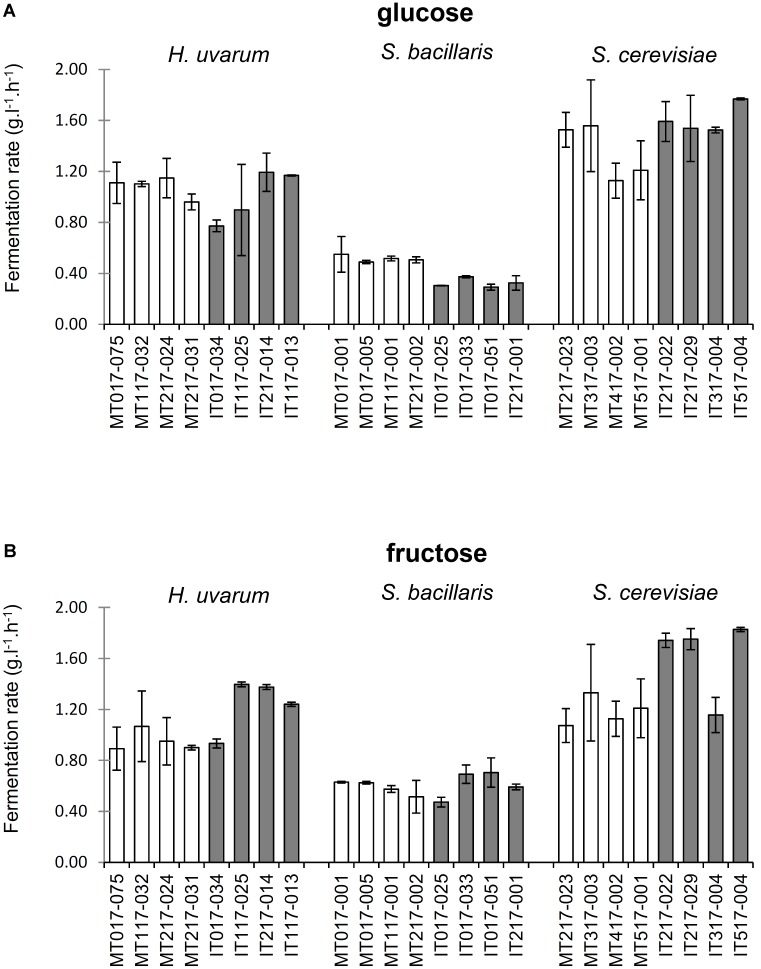
Fermentation rates of *H. uvarum, S. bacillaris* and *S. cerevisiae* isolates from Malbec and Isabella ecosystems. Fermentation rate values (g.l^-1^.h^-1^) were obtained by linear regression of culture weight loss values for the first 24 h of cultures of the indicated isolates in media supplemented with either glucose **(A)** or fructose **(B)**. Experiments were performed in duplicate and bars represent the linear regression error of the 95% confidence band.

A simple microtube assay was designed to explore possible phenotypic differences in glucose versus fructose utilization among the *H. uvarum, S. bacillaris*, and *S. cerevisiae* isolates from the Malbec and Isabella ecosystems. Remarkable differences in the fermentation rates between the three analyzed yeast species were observed at initial stages of fermentation (**Figure 4**). Based on this observation, the initial (i.e., 24 h) fermentation rate phenotype was used to compare the *H. uvarum, S. bacillaris*, and *S. cerevisiae* isolates. Results from these studies showed a discrete heterogeneity in fermentation rate phenotypes, both in glucose- and fructose-containing media, for the various isolates analyzed (**Figure 5**). *H. uvarum* isolates from Isabella showed slightly higher fermentation ability when grown in YP medium containing fructose versus glucose as the major carbon source (**Figure 5**). Interestingly, the average initial fermentation rate phenotype of *S. bacillaris* isolates from Malbec and Isabella were ∼1.5- and ∼1.9-fold higher in fructose than in glucose, respectively (**Figure 5**).

### Microsatellite Genotyping of *S. cerevisiae* Isolates From Malbec and Isabella Ecosystems

Microsatellite genotyping was used to determine if the *S. cerevisiae* isolates from the Malbec and Isabella ecosystems were genetically related. Results from the analyses of seven highly informative microsatellite loci (Legras et al., 2005) are shown in **Table 3**. Loci C3 and C6 were non discriminant and M3 and M4 isolates could not be differentiated in the analysis. Results from **Table 3** show that Malbec and Isabella fermenting musts harbor a genetically diverse population of *S. cerevisiae* strains.

**Table 3:** Genotypes of *S. cerevisiae* isolates from Malbec and Isabella ecosystems.

Isolate^1^	*S. cerevisiae* microsatellite^2^						
	
	AAT1	AAT2	AAT3	C3	C6	YPL009c	YOR267c
M1	1	1	2	1	1	3	2
M2	1	1	1	1	1	1	1
M3	1	1	2	1	1	2	2
M4	1	1	2	1	1	2	2
I1	4	1	2	1	1	3	2
I2	6	2	ND	1	1	2	ND
I3	5	1	2	1	1	3	3
I4	4	1	4	1	1	2	1

*^1^S. cerevisiae isolates from Malbec (M) and Isabella (I) are: M1 (MT217-023), M2 (MT317-003), M3 (MT417-002), M4 (MT517-001), I1 (IT217-022), I2 (IT217-029), I3 (IT317-004), and I4 (IT517-004) (see **Table 2**); ^2^Microsatellite loci are described in Legras et al. (2005); Numbers identify the different alleles recognized for each microsatellite locus. ND, not determined.*

## Discussion

Spontaneously fermenting grape musts constitute rich microbial ecosystems, harboring a remarkable diversity of yeast species. The assembly and evolution of this microbiota, from grape development to the end of must fermentation, is conditioned by the intrinsic biological properties of the grapevine, geographic and climatic conditions at the vineyard, agricultural practices and winemaking procedures (Bokulich et al., 2014; Knight et al., 2015; Jara et al., 2016; Drumonde-Neves et al., 2017).

We have recently proposed that some yeast species may be specifically associated with some *Vitis* species (Raymond Eder et al., 2017). Eventually, different *Vitis* species may harbor specific yeast communities (i.e., yeast species and/or strains of a given yeast species) even in neighboring *Vitis* ecosystems. In this work we explored this hypothesis by studying yeast isolates, from neighboring Malbec (*V. vinifera* L.) and Isabella (*V. labrusca* L.) vineyards, representative of the three major species recognized in Isabella (i.e., *H. uvarum, S. bacillaris*, and *S. cerevisiae*) (Raymond Eder et al., 2017). *H. uvarum* was the predominant non-*Saccharomyces* species in the Malbec and Isabella ecosystems, both at early and middle stages of fermentation. In a previous work, we identified *S. bacillaris* as the main yeast species present at early stages of fermentation of Isabella grapes harvested in the same geographic region (i.e., vintage 2015) (Raymond Eder et al., 2017). A similar predominance of either *H. uvarum* or *C. stellata* (reclassified to *S. bacillaris*; Csoma and Sipiczki, 2008; Duarte et al., 2012), in consecutive vintages in the same geographic region, has been reported (Beltran et al., 2002). In addition to *H. uvarum*, a variety of non-*Saccharomyces* species were isolated at early stages of Isabella must fermentation. This diversity quickly decreased between *t0* and *t24* and three main yeast species (i.e., *H. uvarum, C. californica*, and *C. hellenica*) were recognized following 1 day of fermentation. In fermenting Malbec grape must, on the other hand, the great diversity of yeast species found at the beginning of fermentation continued at *t24* and *t48*, when *S. cerevisiae* species started to develop.

A total of seventeen different yeast species were isolated from both Malbec and Isabella musts at early stages of fermentation. Although all of these yeast species have previously been described in winemaking environments, their relative contribution to the different neighboring *Vitis* ecosystems analyzed in this work varied. For example, *H. vineae, M. pulcherrima*, and *T. delbrueckii*, yeast species commonly found in *V. vinifera* L. grape musts (Jolly et al., 2006), were isolated only from the Malbec ecosystem. The relatively low number of isolates (i.e., 80 isolates from each Malbec and Isabella), however, does not allow to conclude if these yeast species have preferential association with the Malbec versus the Isabella ecosystem. Interestingly, *M. pulcherrima* was not identified in fermenting Isabella must from grapes analyzed in this work nor in grapes harvested from the same vineyards in a previous vintage (Raymond Eder et al., 2017). In addition, *M. pulcherrima* was identified in *V. labrusca* L. grapes from the Azores Archipelago, but only with very low frequency (1.08% of the total isolates) (Drumonde-Neves et al., 2016). On the other hand, the rare yeast species *C. californica*, isolated from Isabella spontaneously fermenting must in the vintage of year 2015 in Colonia Caroya (Raymond Eder et al., 2017), was identified again in the same Isabella ecosystem in this work (i.e., vintage 2017). Moreover, *C. californica* was not found among a total of 150 isolates from the analyzed Malbec ecosystem. Taken together, these observations suggest that *M. pulcherrima* and *C. californica* could have apparent selective and/or preferential association with *V. vinifera* L. and *V. labrusca* L. ecosystems, respectively. However, although *C. azymoides* was originally found associated with fermenting must only from *V. labrusca* L. grapes (Drumonde-Neves et al., 2016; Raymond Eder et al., 2017), this yeast species was also recognized in the Malbec ecosystems studied in this work. Remarkably, *C. azymoides* has not previously been recognized in the extensive worldwide studies performed on the yeast microbiota of *V. vinifera* L. grapes and musts. Therefore, we hypothesize that *C. azymoides* isolates may be limited to some specific *terroirs*, and/or its presence in our Malbec samples may be dependent on the close location of *V. vinifera* L. and *V. labrusca* L. vineyards in Colonia Caroya.

Phenotypic analyses of H_2_S production showed a remarkable diversity among the analyzed *S. cerevisiae* isolates from Malbec and Isabella. Microsatellite genotyping of these *S. cerevisiae* isolates showed that, with the exception of isolates M3 and M4, they correspond to genetically different strains. Interestingly, Isabella’s *S. cerevisiae* isolates I2 and I3, which are genetically different, were the lowest producers of H_2_S, even when compared with the industrial strain EC1118. Additional characterization of a larger number of *S. cerevisiae* isolates could indicate if medium and low H_2_S producer strains are preferentially associated with the Malbec and Isabella ecosystems, respectively. *H. uvarum* and *S. bacillaris* isolates were low and high producers of H_2_S, respectively. Although the observed phenotypes suggested genetic heterogeneity among the analyzed *H. uvarum* and *S. bacillaris* isolates, no specific association of the isolates with their Malbec or Isabella ecosystems was observed.

*Hanseniaspora uvarum* and *S. bacillaris* isolates, from both the Malbec and Isabella ecosystems, showed a relatively low tolerance to ethanol. Although *H. uvarum* and *S. bacillaris* have been found at final stages of spontaneous fermentation of *V. vinifera* L. musts (Combina et al., 2005; Tofalo et al., 2011; Aponte and Blaiotta, 2016; Tristezza et al., 2016), low tolerance to ethanol of *S. bacillaris* from fermenting Isabella grape must has been described (Raymond Eder et al., 2017). On the other hand, *S. cerevisiae* isolates from the Malbec or Isabella musts showed some mild differences in tolerance to ethanol. Ethanol tolerance of the Malbec *S. cerevisiae* isolates was similar among isolates obtained at either medium or advanced stages of fermentation (not shown). Interestingly, ethanol yield in completely fermented Isabella grape must was lower than expected. This phenomenon, which is not observed for Malbec or other *V. vinifera* L. grape musts from Colonia Caroya (Córdoba, Argentina), is typically observed in spontaneously fermented Isabella grape musts from this geographic region, regardless of the vintage (Raymond Eder et al., 2017).

Additional evidence on the phenotypic diversity of yeast species isolated from the Malbec and Isabella ecosystems was obtained from the analysis of their fermentation profiles in media containing either glucose or fructose as the main carbon source. Interestingly, some *S. cerevisiae* isolates appear to have a slightly higher fermentation rate in fructose than in glucose media, which was unexpected given the glucophilic character of this yeast species. Also interestingly, *S. bacillaris* isolates from the Malbec ecosystem showed higher fermentation rates in media containing glucose than *S. bacillaris* isolates from Isabella.

Finally, our results show a remarkable biodiversity among main yeasts isolated from two different neighboring *Vitis* ecosystems and provide preliminary evidence on the potential specific association between *Vitis* species and yeast species and strains. The dynamics of specific yeast populations during spontaneous fermentation could translate into specific organoleptic and sensory characteristics of the final wines, dependent on each *Vitis* species. As shown in this work, Isabella and/or other non-conventional *Vitis* ecosystems may harbor yeast species and/or strains with unique metabolic properties which may not be present in *V. vinifera* L. Thus, non-*vinifera* ecosystems may offer an opportunity to look for valuable *Saccharomyces* and non-*Saccharomyces* strains of potential relevance for the winemaking industry.

## Author Contributions

MR, FC, and AR made fundamental contributions to the conception and design of the work, contributed to the acquisition, analysis, and interpretation of data, and drafted the work and revised it critically for intellectual content. All authors approved the final version of the manuscript to be submitted for publication and agreed to be accountable for all aspects of the work in ensuring that questions related to the accuracy and integrity of any part of the work are appropriately investigated and resolved.

## Conflict of Interest Statement

The authors declare that the research was conducted in the absence of any commercial or financial relationships that could be construed as a potential conflict of interest.

## Footnotes

1 https://blast.ncbi.nlm.nih.gov/Blast.cgi
